# PREVALENCE AND RISK FACTORS OF PRESENTEEISM DURING THE COVID-19 PANDEMIC IN KOREA: ANALYSIS OF POPULATION-BASED PANEL DATA

**DOI:** 10.13075/ijomeh.1896.02490

**Published:** 2024

**Authors:** Inmyung Song

**Affiliations:** Kongju National University, College of Nursing and Health, Gongju, South Korea

**Keywords:** absenteeism, presenteeism, risk factor, prevalence, chronic disease, COVID-19

## Abstract

**Objectives:**

Defined as attending work while ill, presenteeism is highly prevalent and has negative consequences such as reduced productivity and lower well-being for workers. This study aims to estimate the prevalence of presenteeism among Korean workers during the COVID-19 pandemic and identify the risk factors.

**Material and Methods:**

Using data extracted from the second wave of the Korea Health Panel Survey in 2019–2021, this study assessed experience of presenteeism and the number of presenteeism days as the dependent variable. Independent variables included gender, age group, educational level, employment status, household income quartile, and the presence of chronic conditions. Two generalized estimating equation models were used.

**Results:**

In 2019, 30.6% of participants reported experiencing presenteeism; this decreased to 17.9% in 2020 and 13.5% in 2021. The mean number of presenteeism days decreased from 2.30 days in 2019 to 1.46 days in 2020 and to 1.04 days in 2021. The participants were less likely to experience presenteeism in 2020 and 2021 than in 2019 (OR = 0.48 and OR = 0.36, respectively, p < 0.001). The risk of presenteeism is higher among women, younger age groups, participants with regular employment or on-contract positions, lower income, and chronic conditions than their counterparts. The number of presenteeism days significantly decreased in 2020 and 2021 (β = −0.44 and −0.78, respectively, p < 0.001).

**Conclusions:**

Workers were less likely to experience presenteeism in 2020 and 2021 than in 2019, and the number of presenteeism days decreased during the COVID-19 pandemic. The risk of presenteeism was associated with worker characteristics. Int J Occup Med Environ Health. 2024;37(6):617–29

## Highlights

- Workers in Korea were less likely to experience presenteeism during the pandemic.
- The number of presenteeism days decreased over time.
- The risk of presenteeism was associated with worker characteristics.

## INTRODUCTION

Presenteeism is defined as “attending work while ill” [1]. The dynamic model of presenteeism and absenteeism developed by Johns [1] outlines key variables that trigger and influence the choice of behavior. According to the model, regular attendance of work is disrupted by a health event, whether acute or chronic, and the choice of presenteeism over absenteeism is influenced by worker characteristics such as gender, work attitude, and personality. Presenteeism is further influenced by context factors such as job insecurity, workplace culture, and policies. Presenteeism has many negative consequences, such as mental distress including anxiety and depression [2,3], errors [2] and reduced productivity at work [4], and lower levels of well-being [5]. Furthermore, prospective studies have linked presenteeism to an increased risk of deteriorated general health [6] and future absenteeism [7]. Presenteeism can be particularly problematic for individuals with infectious diseases, as it may endanger coworkers and the public [8]. In occupational settings like healthcare, where presenteeism is more common, it poses significant risks to others [9].

According to a meta-analysis, the prevalence of presenteeism is very high: 35–97% [8]. The prevalence of presenteeism associated with respiratory infectious diseases also remains substantial: 14.1–55% [10]. While much is known about the prevalence of presenteeism worldwide, little is known in the Korean context. So far, it has been documented that 21.7% of wage earners in Korea went to work while ill in 2011 [11]. In 2017, 15.9% of full-time salaried workers in Korea reported experiencing presenteeism [12]. According to a nationwide survey in 2020, 9.1% of employees in Korea could not take sick leave during the COVID-19 pandemic [13]. These studies are based on cross-sectional data [11,12], and the existing literature does not indicate if presenteeism has changed over time in Korea. Therefore, this study aims to estimate the changing prevalence of presenteeism among Korean adults from the pre-pandemic period to post-pandemic era using population-based panel survey data.

Due to the high prevalence of presenteeism and its negative consequences, growing attention has been paid to its predictors [14,15]. The risk of presenteeism has been associated with characteristics at multiple levels. First, the characteristics of workplace, such as sick pay policy and presenteeism culture, are identified as risk factors [8,10]. The absence of sick pay policies in the workplace can compel workers to attend work despite being ill [8]. Moreover, organizational culture is a risk factor of presenteeism, which is prevalent among particular professions like healthcare [16]. Job insecurity is also associated with an increased risk of presenteeism [17]. During the COVID-19 pandemic, Japanese workers in corporate settings were more likely to go to work even with the onset of cold-like symptoms than self-employed workers [18]. In addition, the risk of presenteeism is associated with workers’ sociodemographic characteristics. Individual risk factors for presenteeism include young age, female gender, and having a chronic disease [10,19,20]. During the COVID-19 pandemic, unmarried Japanese workers were more likely to work while being sick than their married counterparts [21].

Presenteeism presents a significant challenge to individuals and societies during the pandemic, as it increases the risk of pathogen transmission. Therefore, studying presenteeism in the context of the COVID-19 pandemic is particularly important. Furthermore, there are indications that workplaces globally have become more accepting of remote work in the aftermath of the pandemic [22], which could affect the likelihood of presenteeism in the workplace. Presenteeism has been shown to be significantly associated with job insecurity, which was prompted by the pandemic in some industries, such as hospitality [23].

Unlike abundant information available on the predictors of presenteeism worldwide, understanding is limited in the Korean context. So far, presenteeism among Korean workers has been shown to be associated with socioeconomic characteristics, such as education level, income, and employment type (full-time vs. part-time) [11]. However, the findings are based on univariate analysis [11], calling for a closer examination of influencing factors. Therefore, the objectives of this study are to estimate the prevalence of presenteeism in Korea and to identify individual factors influencing presenteeism, focusing on sociodemographic and health-related characteristics among Korean workers. Additionally, this study can fill the gap in the current understanding of the changes in the trend of presenteeism before and after the COVID-19 pandemic by utilizing longitudinal data. The hypothesis proposed was that presenteeism in Korea is associated with workers’ socioeconomic characteristics, including gender, age, education level, income, and employment status, as well as chronic conditions.

## MATERIAL AND METHODS

### Data

Data were extracted from the second wave of the Korea Health Panel Survey (KHPS) conducted in 2019–2021. The Korea Health Institute for Health and Social Affairs, in collaboration with the National Health Insurance Service, has conducted this population-based panel survey since 2008. The survey uses a 2-stage stratified cluster sampling design to select a panel of households. All members aged ≥19 years in the selected households are surveyed using a structured questionnaire. The panel survey was conducted in August–December 2019 (N = 18 630), June–October 2020 (N = 16 587), and March–July 2021 (N = 14 844) [24]. The KHPS does not, in principle, allow for sample replacement. However, after the study period, efforts were made to restore households that had dropped out by 2021.

The inclusion criteria for the samples analyzed in this study are wage earners and individuals ≥30 years of age. Respondents were not excluded based on other characteristics such as gender, marital status, or health status. The age cut-off was used because, in Korea, many young adults do not begin working until their late 20s due to extended schooling and/or mandatory military service. The sample sizes analyzed were 3539 for 2019, 3509 for 2020, and 3585 for 2021.

### Measures of presenteeism

In the KHPS, participants were asked if they had attended work in the past year despite being sick. If they answered yes, they were then asked how many days they had worked while ill [24]. This study examined 2 measures of presenteeism available in the KHPS data. The first is a binary variable indicating whether the participant reported to work while being ill. The second is a count variable indicating the frequency of presenteeism.

### Explanatory variables

Based on a review of the literature [10,19–21,25], and the availability of information in the KHPS dataset, this study included worker characteristics such as gender (male and female), age, educational level, marital status, employment status, household income quartile, and the presence of chronic conditions. Age was grouped into 4 categories (30–39 years, 40–49 years, 50–59 years, and ≥60 years). Educational level was categorized into 3 groups (middle school or lower, high school, and college or higher). Marital status was divided into 3 categories (married, divorced/separated/widowed, and single). Employment status was divided into 3 categories (regular employment, on-contract employment, and day labor). In the KHPS, information on chronic conditions was collected, including whether the respondents had hypertension, diabetes mellitus, liver diseases, musculoskeletal disorders, cancer, heart disease, cerebrovascular diseases, respiratory diseases, and mental disorders. The variable for chronic conditions was dichotomized into “yes” and “no.”

### Statistical analysis

The characteristics of participants were described using frequency and percentage for each year between 2019–2021. The prevalence of presenteeism was calculated as the percentage of participants who experienced presenteeism each year. This percentage is adjusted for sampling weight. The weight is the inverse of the sampling probability, which varies for subgroups in the complex survey design [26]. The weighting accounted for the issue of attrition inherent in the panel survey.

The frequency and percentage of participants who experienced presenteeism in 2019 were described by socioeconomic and health-related characteristics. The Rao-Scott χ^2^ test, recommended for complex survey data, was used to test for significant differences among categories [27]. The mean number of presenteeism days was calculated and plotted by year for all workers and for those who answered “yes” to experiencing presenteeism. Since the number of presenteeism days showed a non-normal distribution, the Kruskal-Wallis test was used to test for differences among groups [28]. A power analysis was conducted using the POWER procedure in Statistical Analysis System (SAS) to determine if the sample size was sufficient for this study. With a significance level of α = 0.05 and statistical power equal 0.80, the minimum sample size required per group was N = 64 to detect a difference in the number of presenteeism days between individuals with and without chronic conditions. This indicates that the sample size in this study is more than adequate to test the study hypothesis. To examine the risk factors for presenteeism, 2 regression models were used. First, logistic regression analysis was conducted with the experience of presenteeism as the dependent variable. A generalized estimating equation (GEE) model was also used to account for the correlation in panel data. This was implemented using the GENMOD procedure in SAS, specifying the options for binomial distribution and log link function. The first model included all participants who answered the question about presenteeism experience. Adjusted odds ratio (OR) and 95% confidence intervals (CI) were calculated. Quasi-likelihood under the independence model information criterion (QIC) was examined as a GEE fit criterion.

For those who reported experiencing presenteeism, the follow-up analytical strategy was to estimate the impact of predictors on the number of presenteeism days. Since the number of presenteeism days is a right-skewed count variable following a Poisson distribution, another GEE model was conducted using the GENMOD procedure in SAS with the Poisson distribution option. In this study, the GEE approach was chosen over mixed effect modelling to address correlation in the longitudinal data. This choice was made because GEE is considered more appropriate for estimating the average effect of independent variables on the outcome variable in the population [29].

All statistical analyses were performed using SAS v. 9.4 (Cary, NC, USA). The Institutional Review Board of Kongju National University, South Korea, approved the study protocol and waived the requirement for informed consent (reference No. KNU_IRB_2024-056).

## RESULTS

**Figure 1.**
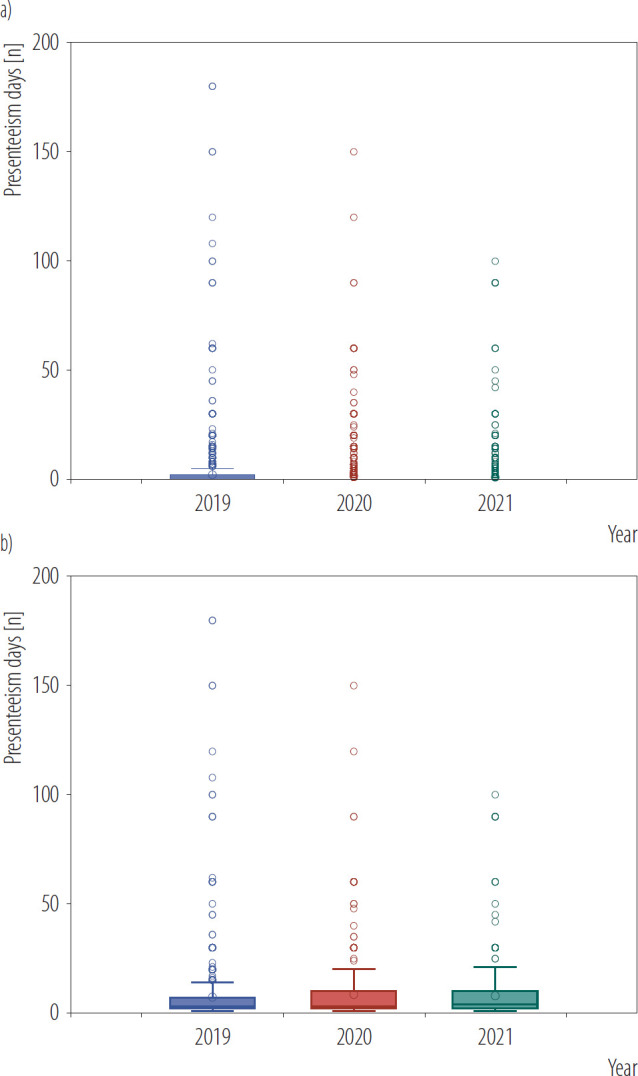
Box plots of presenteeism days by year a) among all workers, b) among workers who answered yes to presenteeism experience, data extracted from the second wave of the Korea Health Panel Survey (KHPS) conducted in 2019–2021, South Korea

A total of 3539, 3509, and 3585 participants were wage earners in 2019, 2020, and 2021, respectively (Table 1). In 2019, 1863 (54.3%) were men, 1039 (24.9%) were aged ≥60 years, and 2560 (66.9%) were currently married. Additionally, 1645 (55.1%) had a college or higher education and 1975 (61.7%) had regular employment. A total of 1237 (27.6%) had chronic conditions. In 2019, 30.6% of participants reported experiencing presenteeism; this decreased to 17.9% in 2020 and 13.5% in 2021. The mean number of presenteeism days decreased from 2.3 days in 2019 to 1.5 days in 2020 and to 1.0 days in 2021 (Table 1 and Figure 1).

**Table 1.** Characteristics of study participants, wage earners, data extracted from the second wave of the Korea Health Panel Survey (KHPS) conducted in 2019–2021, South Korea

Variable	Participants					
	2019 (N = 3539)		2020 (N = 3509)		2021 (N = 3585)	
	n (%)	M±SE	n (%)	M±SE	n (%)	M±SE
Gender						
male	1863 (54.3)		1855 (55.4)		1844 (54.0)	
female	1676 (45.7)		1654 (44.6)		1741 (46.0)	
Age						
30–39 years	780 (30.2)		808 (31.2)		792 (30.7)	
40–49 years	886 (26.5)		864 (26.3)		846 (25.0)	
50–59 years	834 (24.9)		822 (24.7)		844 (25.2)	
≥60 years	1039 (18.5)		1015 (17.8)		1103 (19.1)	
Education level						
middle school or lower	645 (11.3)		614 (10.0)		602 (9.1)	
high school	1249 (33.6)		1244 (34.5)		1288 (34.9)	
college or higher	1645 (55.1)		1651 (55.5)		1695 (56.0)	
Marital status						
currently married	2560 (66.9)		2519 (67.2)		2504 (64.1)	
divorced/separated/widowed	430 (10.6)		444 (10.7)		458 (11.0)	
single	548 (22.5)		546 (22.1)		623 (24.9)	
Employment status						
permanent	1975 (61.7)		1998 (62.9)		1884 (58.4)	
on contract	1074 (26.9)		1098 (28.0)		1204 (30.7)	
day labor	490 (11.4)		413 (9.1)		497 (10.9)	
Household income						
first (lowest) quartile	870 (20.2)		874 (19.6)		897 (18.6)	
second quartile	871 (24.3)		876 (23.4)		895 (24.0)	
third quartile	870 (26.8)		876 (26.5)		896 (26.0)	
fourth (highest) quartile	870 (28.7)		875 (30.5)		896 (31.5)	
Chronic condition						
yes	1237 (27.6)		1331 (30.7)		1457 (33.1)	
no	2302 (72.4)		2178 (69.3)		2128 (66.9)	
Experience of presenteeism						
yes	1043 (30.6)		599 (17.9)		465 (13.5)	
no	2496 (69.4)		2910 (82.1)		3120 (86.5)	
Presenteeism days [n]		2.30±0.21		1.46±0.15		1.04±0.12

Percentage is adjusted for sampling weight.

The percentage of participants who experienced presenteeism in 2019 differed between men and women and among different age groups (p < 0.001) (Table 2). The percentage also differed among various educational levels and employment statuses (p < 0.05). Additionally, the number of presenteeism days differed between men and women, as well as among different age groups, educational levels, and employment statuses (p < 0.001).

**Table 2.** Experience of presenteeism and number of presenteeism days by sociodemographic and health-related characteristics in 2019, data extracted from the second wave of the Korea Health Panel Survey (KHPS) conducted in 2019–2021, South Korea

Variable	Participants (N = 3539)		p	Presenteeism days		p
	total [n]	experiencing presenteeism [n (%)]		mean rank	Kruskal-Wallis χ^2^	
Gender						
male	1863	501 (26.9)	<0.001	1725	11.8	<0.001
female	1676	542 (32.3)		1820		
Age						
30–39 years	780	292 (37.4)	<0.001	1841	57.2	<0.001
40–49 years	886	320 (36.1)		1879		
50–59 years	834	244 (29.3)		1759		
≥60 years	1039	187 (18.0)		1610		
Education level						
middle school or lower	645	146 (22.6)	0.011	1667	18.9	<0.001
high school	1249	353 (28.3)		1748		
college or higher	1645	544 (33.1)		1827		
Marital status						
currently married	2560	745 (29.1)	0.664	1765	0.6	0.740
divorced/separated/widowed	430	131 (30.5)		1798		
single	548	166 (30.3)		1771		
Employment status						
permanent	1975	633 (32.1)	0.011	1811	20.2	<0.001
on contract	1074	308 (28.7)		1760		
day labor	490	102 (20.8)		1626		
Household income						
first (lowest) quartile	870	249 (28.6)	0.997	1738	0.1	0.991
second quartile	871	256 (29.4)		1738		
third quartile	870	264 (30.3)		1749		
fourth (highest) quartile	870	263 (30.2)		1739		
Chronic condition						
yes	1237	338 (27.3)	0.177	1749	1.2	0.271
no	2302	705 (30.6)		1781		

The Rao-Scott χ^2^ test and the Kruskal-Wallis test were used to test for differences in the experience of presenteeism and the number of presenteeism days, respectively.

The participants were less likely to experience presenteeism in 2020 and 2021 than in 2019 (OR = 0.48, 95% CI: 0.43–0.54, p < 0.001 for 2020; OR = 0.36, 95% CI: 0.32–0.40, p < 0.001 for 2021) (Table 3). Compared to men, women had an increased risk of presenteeism (OR = 1.31, 95% CI: 1.17–1.47, p < 0.001). The risk of presenteeism was higher in younger age groups than in the ≥60 years old group:

– OR = 2.35, 95% CI: 1.87–2.93, p < 0.001 for 30–39 years;
– OR = 2.26, 95% CI: 1.87–2.73, p < 0.001 for 40–49 years;
– OR = 1.59, 95% CI: 1.34–1.90, p < 0.001 for 50–59 years.

**Table 3.** Regression analysis on the experience of presenteeism (model 1: N = 10 565, QIC = 10 040) and the number of presenteeism days (model 2: N = 10 565, QIC = 535), data extracted from the second wave of the Korea Health Panel Survey (KHPS) conducted in 2019–2021, South Korea

Variable	Model 1			Model 2		
	OR	95% CI	p	estimate	SE	p
Year (ref. 2019)						
2020	0.48	0.43–0.54	<0.001	−0.44	0.10	<0.001
2021	0.36	0.32–0.40	<0.001	−0.78	0.11	<0.001
Gender (ref. male)						
female	1.31	1.17–1.47	<0.001	0.16	0.10	0.090
Age (ref. ≥60 years)						
30–39 years	2.35	1.89–2.93	<0.001	0.65	0.19	0.001
40–49 years	2.26	1.87–2.73	<0.001	0.68	0.19	<0.001
50–59 years	1.59	1.34–1.90	<0.001	0.45	0.15	0.004
Education level (ref. middle school or lower)						
high school	0.96	0.80–1.16	0.699	−0.10	0.16	0.515
college or higher	1.00	0.81–1.22	0.974	0.03	0.20	0.883
Marital status (ref. single)						
currently married	1.07	0.90–1.28	0.452	0.08	0.13	0.563
divorced/separated/widowed	1.30	1.02–1.65	0.032	0.12	0.18	0.497
Employment status (ref. day labor)						
permanent	1.54	1.27–1.87	<0.001	0.23	0.18	0.203
on contract	1.44	1.19–1.74	<0.001	0.26	0.17	0.122
Household income (ref. fourth [highest] quartile)						
first (lowest) quartile	1.26	1.05–1.50	0.012	0.47	0.16	0.003
second quartile	1.13	0.97–1.32	0.114	0.21	0.13	0.092
third quartile	1.14	0.98–1.32	0.083	0.26	0.14	0.057
Chronic condition (ref. no)						
yes	1.44	1.27–1.64	<0.001	0.75	0.12	<0.001

QIC − quasi-likelihood under the independence model information criterion.

Divorced, separated, and widowed participants had significantly higher odds of experiencing presenteeism than single participants (OR=1.30, 95% CI: 1.02–1.65, p < 0.05). Participants with regular employment (OR = 1.54, 95% CI: 1.27–1.87, p < 0.001) and those in on-contract positions (OR = 1.44, 95% CI: 1.19–1.74, p < 0.001) had an increased likelihood of presenteeism compared to those in day-labor employment. The lowest income quartile was associated with an increased risk of presenteeism compared to the highest income quartile (OR = 1.26, 95% CI: 1.05–1.50, p < 0.05). Workers with chronic conditions are more likely to experience presenteeism than those without chronic conditions (OR = 1.44, 95% CI: 1.27–1.64, p < 0.001).

The number of presenteeism days decreased in 2020 and 2021 (β = −0.44 and −0.78, respectively, p < 0.001). Younger workers had fewer presenteeism days than workers aged ≥60 years old (β = 0.7 for 30–39 years, 0.7 for 40–49 years, and 0.5 for 50–59 years, respectively, p < 0.01). Workers in the lowest income quartile had more presenteeism days than those in the highest quartile (β = 0.5, p < 0.01). Workers with chronic conditions had more presenteeism days than those without chronic conditions (β = 0.8, p < 0.001).

## DISCUSSION

In this study, the prevalence of presenteeism among Korean workers decreased from 30.6% in 2019 to 17.9% in 2020, and further to 13.5% in 2021. This decline may be attributed, in part, to the increased adoption of teleworking during the COVID-19 pandemic [30]. Prior to the pandemic, telecommuting was utilized by only a small proportion of Korean workers, which was substantially less than in Western nations like the U.S. and Germany; however, the acceptance of home-based teleworking appears to have markedly increased during the pandemic [31,32]. There are also other potential factors that could have contributed to the decrease in presenteeism during the COVID-19 pandemic, such as the fear of transmitting the virus by going to work and society's decreasing acceptance of working while ill [33].

This study identified individual factors, sociodemographic characteristics, and the presence of chronic diseases as influencing the likelihood of presenteeism. Notably, individuals in the lowest income quartile were more likely to experience presenteeism than those in the highest income quartile. Existing studies in Korea and other countries suggest that financial insecurity contributes to the tendency to attend work despite being ill [25,34]. A study based on 2014 data showed that Korean men who felt insecure about their jobs were more likely to report presenteeism [25]. Fear of jobloss increases the risk of presenteeism among healthcare workers in Switzerland [19]. Job insecurity in an unfavorable job market may encourage people to work despite being sick [33]. The U.S. workers who had financial insecurity were more likely to intend to work if they got sick with the severe acute respiratory syndrome coronavirus (SARS-CoV-2) [34].

In this study, regular employees were more likely to engage in presenteeism than day laborers. This may be because regular employees may feel greater pressure to perform, even when unwell. Job demand was a significant correlate of presenteeism and may have been more acutely felt by those in regular employment positions compared to day laborers [35]. Permanent employees in the Finnish government sector have an increased risk of presenteeism compared to employees on fixed-term contracts [17].

Moreover, some professionals’ desire to be present at work while ill might be driven by a strong sense of duty, which is shown to be a significant factor influencing presenteeism [36]. Characteristics of work, such as difficulty of finding staff replacements, can also contribute to the pressure to report to work while ill for workers in regular employment positions [15].

In this study, women are more likely to experience presenteeism than men, which is consistent with the findings from other countries [10,19]. Female physicians in Sweden may be motivated to work while sick due to concerns that others will bear greater workload if they are absent from work [37]. The risk of presenteeism is higher in people whose duties remain if they take sick leave [38]. Female workers may also be more be sensitive to working hour arrangements, which may contribute to their increased risk of presenteeism compared to men [39]. Gender difference may occur because women tend to occupy jobs that are unforgiving of absenteeism. Presenteeism is most common in the education and nursing sectors, which are predominantly occupied by women [38].

This study also shows that young age is an independent risk factor for presenteeism, which is consistent with the findings of previous studies [10,19]. According to a study in Canada, age is the most robust predictor of presenteeism [14]. In particular, younger managers show a higher propensity to work while ill, perhaps due to concerns about their careers [14]. A German study suggests that older workers may be more prone to take sick leave than young workers, when faced with stressful work [40].

In this study, participants with a chronic condition are more likely to report to work while ill than those without a chronic condition, which is consistent with findings from previous studies in other countries [41,42]. According to a study in Sweden, the risk of presenteeism is strongly associated with the presence of a health problem [15]. Patients with diabetes report more days of productivity loss than individuals without diabetes [43]. The risk of presenteeism was higher for individuals at high risk of coronary artery disease and those at high risk of ischemic stroke compared to individuals with a low risk of the respective conditions [44]. Similarly, the severity of asthma was positively associated with presenteeism, which increased as asthma control worsened [45]. Presenteeism is highly prevalent among Swedish workers with back and neck pain [38]. Physical conditions like back problems, gastritis, and allergies are positively associated with the frequency of presenteeism among executive-level public servants in Canada [14]. One interpretation for this association is that symptoms of these conditions can be managed and controlled with medications, which may enable individuals to engage in presenteeism [14]. Individuals with some chronic conditions such as mental disorder may choose to come to work because they find it difficult to prove their illness [46]. The literature suggests that workplace adjustments such as use of assistive technology and modifications of duties and hours could enable patients with chronic conditions to work voluntarily despite being unwell [47].

Based on nationally representative panel survey data, this study demonstrated that both the prevalence of presenteeism and the mean number of presenteeism days have decreased in Korea during the COVID-19 pandemic. Furthermore, this study identified risk factors associated with the experience of presenteeism, which have not been closely examined in the Korean population. The findings of this study will inform policy interventions to target individuals at risk of the potentially harmful behavior of presenteeism and to improve the well-being of workers. Poten tial interventions to reduce presenteeism in the workplace include initiatives aimed at changing the presenteeism culture, improving paid sick leave policies, and providing supervisors with training on how to support employees dealing with mental health issues and other conditions [48]. Future research should examine whether the trend of decreasing presenteeism continues.

Despite the strengths of this study, the results should be interpreted with caution. First, the experience and number of presenteeism days were self-reported and therefore subject to recall bias. Second, the models used in the study are limited by the availability of data in the KHPS dataset. Consequently, this study could not examine the role of workplace characteristics, such as job demand, presenteeism culture and sick leave policies, which could influence presenteeism behaviors. In addition, this study focused solely on chronic conditions, but it would be valuable to examine overall health status in greater depth, as it could also influence presenteeism. Lastly, while this study fills an important gap in the knowledge base regarding presenteeism in Korea, its findings may not be generalizable to other populations.

## CONCLUSIONS

This study showed that workers were less likely to experience presenteeism in 2020 and 2021 than in 2019, and the number of presenteeism days decreased during the COVID-19 pandemic. The risk of presenteeism was positively associated with female sex, younger age, regular employment or on-contract positions, lower income, and chronic conditions. The number of presenteeism days was negatively associated with young age but positively associated with lower income and the presence of chronic conditions. Policy efforts should target individuals who are disproportionately vulnerable to presenteeism. Possible strategies to decrease presenteeism in the workplace involve efforts to shift the presenteeism culture, enhancing paid sick leave policies, and equipping supervisors with training to better support employees managing mental health challenges and other health conditions. In the long term, initiatives to reduce presenteeism in the workplace could enhance employee well-being and productivity. Future research should investigate potential causes for the observed decline in presenteeism during the pandemic, such as the rise in teleworking and changes in workplace policies, and whether this trend is maintained over time.
